# Mining author relationship in scholarly networks based on tripartite citation analysis

**DOI:** 10.1371/journal.pone.0187653

**Published:** 2017-11-08

**Authors:** Feifei Wang, Xiaohan Wang, Siluo Yang

**Affiliations:** 1 School of Economics and Management, Beijing University of Technology, Beijing, China; 2 School of Information and Management, Wuhan University, Wuhan, China; Universidad de las Palmas de Gran Canaria, SPAIN

## Abstract

Following scholars in Scientometrics as examples, we develop five author relationship networks, namely, co-authorship, author co-citation (AC), author bibliographic coupling (ABC), author direct citation (ADC), and author keyword coupling (AKC). The time frame of data sets is divided into two periods: before 2011 (i.e., T1) and after 2011 (i.e., T2). Through quadratic assignment procedure analysis, we found that some authors have ABC or AC relationships (i.e., potential communication relationship, PCR) but do not have actual collaborations or direct citations (i.e., actual communication relationship, ACR) among them. In addition, we noticed that PCR and AKC are highly correlated and that the old PCR and the new ACR are correlated and consistent. Such facts indicate that PCR tends to produce academic exchanges based on similar themes, and ABC bears more advantages in predicting potential relations. Based on tripartite citation analysis, including AC, ABC, and ADC, we also present an author-relation mining process. Such process can be used to detect deep and potential author relationships. We analyze the prediction capacity by comparing between the T1 and T2 periods, which demonstrate that relation mining can be complementary in identifying authors based on similar themes and discovering more potential collaborations and academic communities.

## Introduction

Accurate partners or research followers are imperative in scientific research. Mining deeper author relationships in the academic network involving various significance is achievable, which can help scholars establish potential cooperative or reference relationships. The research visual field can also be expanded, and the research content can be deepened. The establishment of a citation relationship among scholars is mainly based on the correlation of their research contents. If this relationship is deeply mined, potential partners could be found. Given that the citation data were preserved completely and accurately in document database, the processes and results of relationship mining would be feasible and reliable. As a mature quantitative research method in bibliometrics and scientometrics, citation analysis is extensively used in scientific evaluation, scholarly communications, academic behavior analysis, and information retrieval. Author citation analysis mainly includes three types: author co-citation (AC), author bibliographic coupling (ABC), and author direct citation (ADC), which is collectively called “tripartite citation analysis” in this study. For example, in a field, both papers of Authors A and B were cited by the same paper; thus, A and B have a co-citation relationship marked as AC (A, B). Authors C and D both cite the same paper in their respective articles; C and D thus have a bibliographic-coupling relationship marked as ABC (C, D). In addition, Author D cites a paper written by A in his bibliography, or vice versa; thus, D and A have a direct-citation or cross-citation relationship marked as ADC (A, D).

On mining author relationship in scholarly networks based on tripartite citation analysis, two key questions should be addressed.

1. Which bibliographic-coupled or co-cited authors did not collaborate yet or do not cite each other regularly? If we called these relationships as potential communication relationship (PCR), and the latter two as actual communication relationship (ACR), could the discovery and usage of PCR contribute to the achievement of the ACR? Furthermore, how is the quantitative relation of PCR and ACR? This concern is the first point to be investigated in this study.
2. In view of the similarities or diversity among tripartite citation relationships at the author level, how can tripartite relationships be synthetically used in discovering deeper author relationships serving for broader scholarly communication and relevant recommendations? According to these primary relationships, deducing the integrated relationships between Authors A and C, or B and D, even B and C, the association strength in these potential relationships is the second point to be answered in this study.

## Related studies

### Separate study of tripartite citation analysis

AC analysis is the most commonly used method for the empirical analysis of disciplinary paradigm, and has been frequently studied and improved. Some AC analyses have been conducted since Small [1] introduced document co-citation analysis and White and Griffith [2] developed AC analysis. Bibliographic coupling was proposed as early as 1963 [3]. However, author coupling relationship has not gained considerable attention until it was formally proposed and empirically studied by Zhao and Strotmann [4]; the authors named this method ABC analysis, which can be used to complement AC analysis in comprehensively viewing the intellectual structure by mapping the research activities of active authors for a realistic picture of the current state of research in a field.

In comparison with co-citation and bibliographic coupling, direct citation (sometimes also called inter-citation or cross-citation) is a direct citation relationship without a third-party paper. Paper-level direct citation has been used in different scenarios, such as research front detection [5–6], domanial historiography mapping [7], and publication classification [8–9]. Boyack and Klavans [10] found that bibliographic coupling slightly outperforms co-citation analysis and that direct citation is the least accurate science mapping. Shibata et al. [11] revealed that direct citation could detect large and newly emerging clusters earlier, indicating that the research front detection exhibited the best performance, whereas co-citation showed the worst. Numerous studies have focused on journal direct citation; several key research achievements have shown that journal direct citation can reveal the academic influence of journals, as well as the theme evolution and field division of periodicals [12–13]. Direct citation can also be used at the macrolevel, such as citation between subject categories, to build the global map of science [14]. Wang et al. [15] extensively studied ADC analysis, which can be used to determine author relationship from another angle and reveal the knowledge communication and disciplinary structure in scientometrics. This process was then named as ADC analysis by Yang and Wang [16].

### Comparative study of tripartite citation analysis

The three types of citation analysis methods can reveal author relationships in a field in various ways. Some studies have focused on the comparative analysis of these methods, even comparing them with other author co-occurrence network analysis methods, such as co-authorship (CA), word-based author coupling (WAC), and journal-based author coupling (JAC). Related studies are especially represented by Lu, Yan, and Qiu et al. Lu and Wolfram [17] conducted a comparative study of word-based, topic-based, and author co-citation approaches to measure author research relatedness. Findings show that two word-based approaches produced similar outcomes, except in the case in which two authors were frequent co-authors for the majority of their articles, and that topic-based approach produced the most distinctive map. Yan and Ding [18] explored the similarities among six types of scholarly networks (bibliographic-coupling, direct citation, co-citation, topical, co-authorship, and co-word networks) aggregated at institution level; they also detected high similarity between co-citation and direct citation networks. Moreover, the authors recommended the use of hybrid or heterogeneous networks to study research interaction and scholarly communications. Qiu and Dong [19] constructed five types of author co-occurrence networks in the field of information library sciences, such as CA, WAC, JAC, AC, and ABC. In their research, the capabilities of different types of author co-occurrence relationships in revealing scientific structure are compared through hierarchical clustering and correlation analysis by quadratic assignment procedure (QAP) test. ABC analysis also exhibited a significant advantage in revealing discipline structure and presented the highest correlation with other networks. The idea of combining different author co-occurrence networks in scholarly communication and intellectual structure analysis was also proposed.

### Combined study of tripartite citation analysis

The combination of these tripartite citation analysis methods (including AC, ABC, and ADC) has been extensively studied. Small [20] proposed a method for effectively combining them; however, only few researchers have adopted this combined linkage technique at a large scale. Persson [21] and Gómez-Núñez et al. [22–24] have attempted to combine these citation measures in a normalized manner to weigh existing direct citation relationships between articles or journals. According to Persson’s research, direct citations weighted with shared references (bibliographic coupling) and co-citations at the article level could be better applied to domain intellectual structure detection. In addition, citation-based measure calculation and integration (involving co-citation, bibliographic coupling, and cross citation) at the journal level was also proposed and proven in the application of refining the journal classification, improving journal ranking, and further updating the subject classification structure proposed by Gómez-Núñez et al. At the author level, Wang [25] proposed a comprehensive and comparative approach by combining CA, AC, ABC, ADC, and author keyword coupling (AKC), supplemented by social network analysis (SNA), to evaluate the academic impact of the core authors in the field of scientometrics. Existing studies are focused on intellectual structure detection and optimization according to tripartite citation analysis. The assessment of the author scholarly impact by combining various citation analysis is also paid attention in few studies.

### Mining author relationship in scholarly networks

Practical research on the discovery of potential author relationships in communication networks by tripartite citation analysis is limited. Currently, approaches for identifying potential collaboration mainly involve machine-learning techniques, link-prediction techniques, and SNA. Zhang and Yu [26] proposed supervised machine-learning approaches to predict research collaborations by the semantic features in the field of biomedicine and author network topological features, including co-authorship network connectivity, research profile similarity, collective productivity, and seniority. Chen and Fang [27] developed a latent collaboration index model for evaluating the collaboration probability among patent assignees by incorporating two network-related factors (i.e., degree and network distance) and complementary factors (i.e., assignees types, geographical distances, and topic similarities). Guns and Rousseau [28] introduced a method for predicting or recommending high-potential future collaborations based on a combination of link prediction and machine-learning techniques. Daud et al. [29] used discriminative and generative machine-learning techniques for predicting the emerging scholars in a co-author network based on three classes of features (i.e., author, venues, and co-authorship). These studies have focused on the use of combined relationships of direct citation and co-authorship in scholarly networks without considering other relation networks in discovering potential collaboration.

## Data and methodology

### Basic data

Scientometrics is an international journal, launched in 1978. The journal covers all aspects of scientometrics and published 46.31% of scientometrics research paper of the world[30]. Given the Scientometrics Journal as the representative communication channel in the field of scientometrics, the characteristic trends and patterns of the past decades in scientometric research become evident [31]. Bibliographic data from the journal of Scientometrics have formed the main data object in some of recent empirical studies focusing on mapping the intellectual structure[32] or detecting social network community[33] in the field of scientometrics. Therefore, this study also employed bibliographic data that cover all types of documents published in Scientometrics in 1978–2011 and 2011–2015 as representative experimental data object in the field of scientometrics. All data were retrieved from Web of Science (WOS). Data retrieval in the first period was completed in the middle of 2011; the data will be used in deduction and mining. Meanwhile, data retrieval in the second period was completed in the middle of 2015; the data will be used in verifying the results obtained by the first sample.

The first retrieval recalled a total of 2,989 documents, of which 2,982 include author information, 2,815 include references, and 2,812 include both the author information and references. The most prominent contributors are pioneers in most research studies. For example, when evaluating author influence levels, only pioneer authors were considered, which was done by most studies only considered (such as, in uncovering knowledge communication[34], and in revealing implicit relationship [35]) because the cited references only contain the first listed author of the cited document in the database of WOS. Moreover, a complex contribution allocation problem existed when considering all authors in relation analysis. This problem has not been fully solved, which is beyond the scope of this research topic. Thus, only the first authors of each paper were considered in the current study. In counting only the first author in the citation data, the results include 35,796 citations, 16,057 cited authors, and 1,484 citing authors (as first signature identity in the publications). Each author’s name is identified by his surname and first initial only. The second dataset covers 1,318 documents in total, all of which include the author information and 1,308 include references (involving 27,083 valid citations).

### Methodologies

Thus far, a uniform standard for identifying core authors in scientometrics has not been developed. Lotka and Price identified excellent scientists according to the number of their published papers during the study on scientists’ productivity and activity patterns [36]. Garfield treated those authors with high-cited frequency from SCI as excellent scientists [37]. Some scholars also adopted different approaches to evaluate core authors in information science; however, they all considered both the number of published papers and the cited frequency. Therefore, the present study identified 94 authors who have published 5 or more papers and received 10 or more citations as core authors from the first dataset.

AC, ABC, and ADC analyses are used in discovering author relationships with co-citation, bibliographic coupling, and direct citation in scientometrics, respectively. CA and AKC analyses were also complementarily used to discover or verify author relationships in this study. AKC analysis was introduced by Liu et al. [38] and was formally proposed by Liu and Zhang [39]. This method was re-introduced and compared with CA and ABC analyses by Qiu and Wang [40], Liu and Wang [41], Song and Wu [42], and Yang et al. [43]. The AKC analysis is supposed to expand the keyword co-occurrence relationship at the author level; it can also be used to establish author relationships through the keyword coupling strength of authors’ oeuvres. The oeuvres can be used to discover PCRs among authors bound by the same research themes and then describe the knowledge structure of a field or discipline.

The networks of CA and AC were directly constructed by their co-occurrence relationships in the same records. The network of ADC comprised two-way direct citing network between author pairs (i.e., symmetrized by summing the two directional citation values as the total correlation score). The citing and cited links should be equally treated as the direct relationship between author pairs. Thus, the summing symmetrization was selected instead of the lowest or highest value method or even an asymmetrical matrix. However, the symmetrizing processcould be improved by involving the total number of citations and references of authors’ publications to eliminate the effect of the absolute value. Although the original value can reflect an actual situation, the direct citation frequencies must be normalized. However, such normalization could only be done in another study, because researchers have not reached a consensus on which measure is most appropriate for normalization purposes. For ABC and AKC, basic matrixes, including authors*cited reference matrix and authors*keywords matrix, were initially generated and then transformed into ABC and AKC networks via formulas, selecting the minimum method to calculate the coupling strength as suggested by Ma [44]. All of the original co-occurrence matrixes including AC, ADC, ABC, CA and AKC are supplied in the Supporting Information (S1–S5 Tables corresponding to the period of “Before 2011”; S6–S10 Tables corresponding to the period of “After 2011”.)

Co-occurrence analysis and deductive reasoning methods are used in mining deeper and more potential author relationships based on the original tripartite citation analysis. VBA program can process all types of citation analysis data. The final results of author relationship mining will be visualized by the Network Workbench Tool software with the analysis of MST-PathFinder Network Scaling. The use of PathFinder can simplify the network and highlight its important structural features and core associated nodes. This method was used in this study to highlight the visualization of the network and improve map readability.

The QAP is a unique method of measuring relationships in relational data. It compares the value of various corresponding elements in two (or more) squares and gives the Pearson correlation coefficient between two matrixes by comparing the corresponding grid values in each square [45]. A non-parametric test is performed on the coefficients based on the replacement of the matrix data. A comparison on proximity results in this study was conducted using QAP, and the statistics process (including centrality measurement) was annotated in the documentation of Ucinet software [46].

## Study process

### Discovery of PCRs

**Fig 1:**
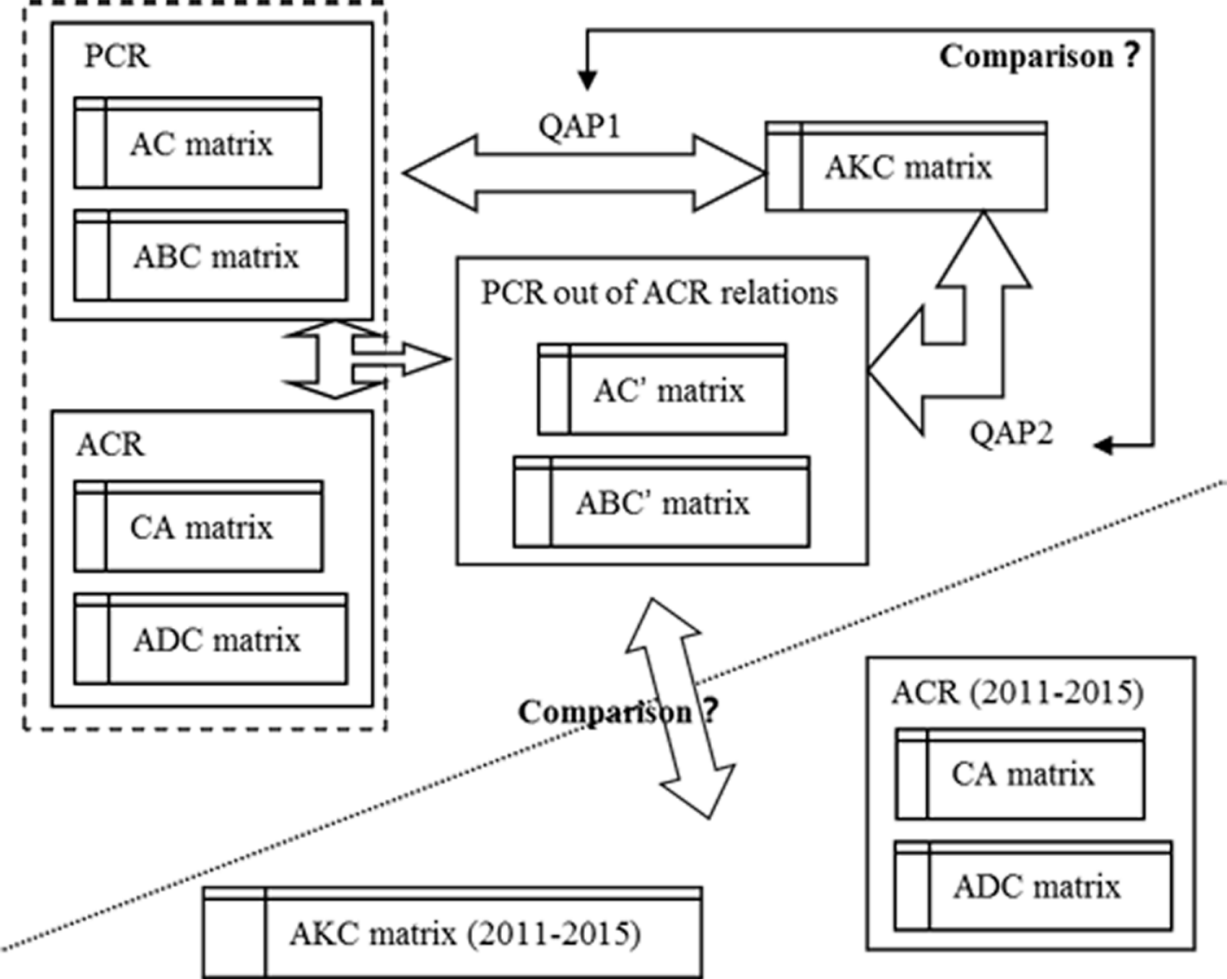
Process and roadmap of PCR discovery.

In this study, five original relation matrixes (including CA, AC, ABC, ADC, and AKC) were first developed. These five matrixes were compared by QAP, and the result was saved and marked as QAP1. Excluding ACR (including CA and ADC matrixes) from PCR (including AC and ABC matrixes), the AC′ and ABC′ matrixes could be obtained (Fig 1). The AC′, ABC′, and AKC matrixes were re-compared, and the result was marked as QAP2. Then, a comparison between QAP1 and QAP2 was performed. AKC is based on the similarity of research themes (can be called “inherent connection”), whereas AC and ABC are based on citation relationships (can be called “exterior connection”). When the inherent and exterior connections are highly consistent with each other, the PCR is assumed to convert into ACR. To test this assumption, the results obtained by PCR from the ACR relationships were compared with the AKC matrix (2011–2015) and ACR matrix (2011–2015).

### Deep relationship mining between author pairs

In this study, the tripartite citation analysis could be applied in deep relationship mining at the author level. To make these relationships comparable, original relation matrixes should be normalized. The normalization method was based on Salton’s cosine similarity measures, which results in similarity values ranging between 0 and 1.

**Fig 2:**
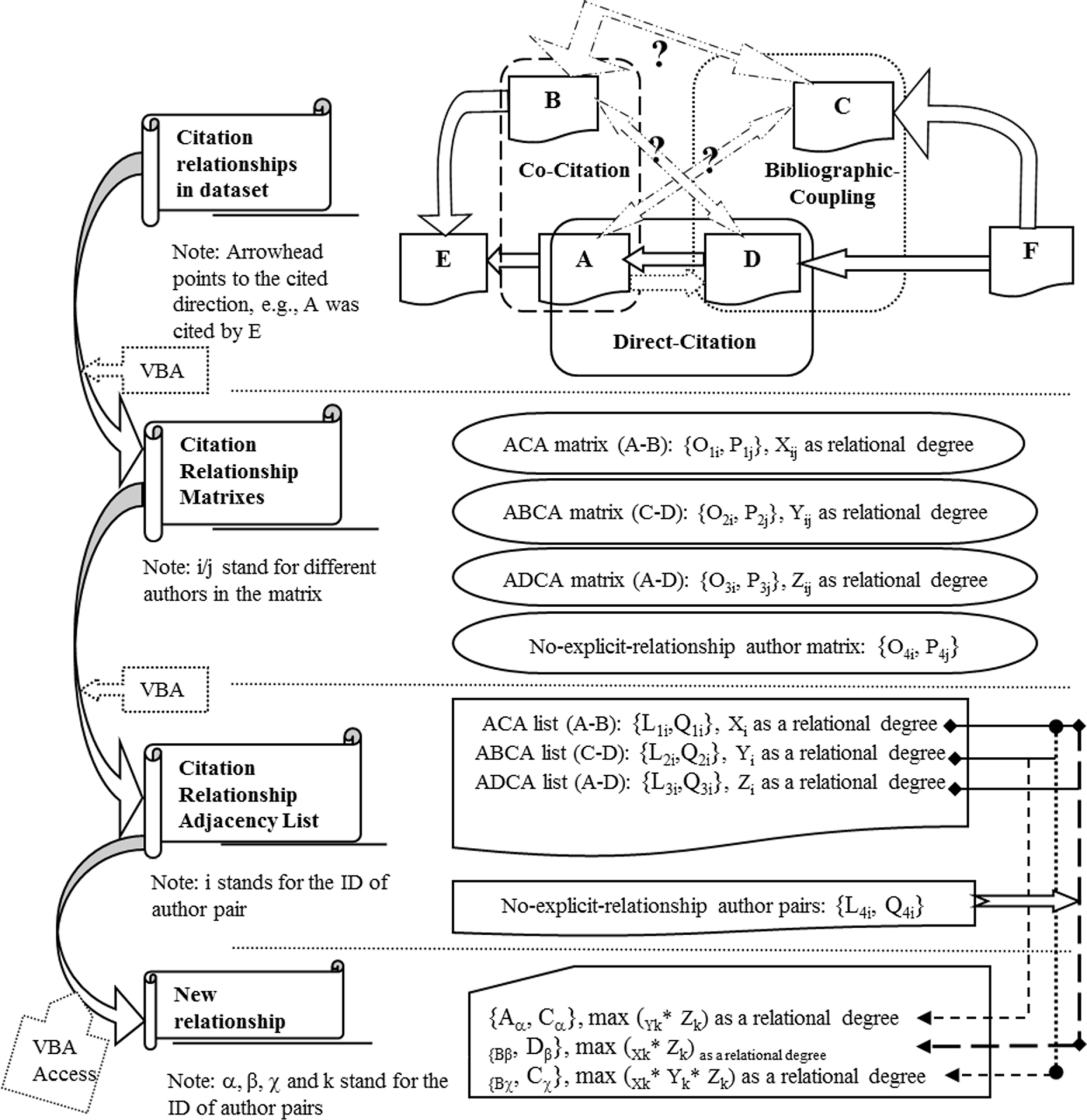
Process and roadmap of relationship mining by tripartite citation analysis.

The following five steps (some of the steps are shown in Fig 2) aid in determining author relationship mining based on tripartite citation analysis, such as “A–C,” “B–D,” and “B–C,” which has been discussed earlier. These steps could also be regarded as the algorithm in relation mining. The implication of each variable A, B, C, and D refers to the author of the matrix, L; Q refers to the relationship between the authors in the adjacency list O; and P refers to the relationship between the authors in the adjacency matrix.

a) *First step: Obtaining the fundamental citation relationship with strength (>0) among the core authors from the original matrixes*.
  Tripartite adjacency matrixes were transformed into the corresponding adjacency list: AC list {L_1i_, Q_1i_} versus matrix {O_1i_, P_1j_}; relational degree X_i_ (i stands for the ID of the author pair) in the list can replace X_ij_ (i/j stand for different authors in the matrix). ABC list {L_2i_, Q_2i_} versus matrix {O_2i_, P_2j_}, and relational degree Y_i_ versus Y_ij_. ADC list {L_3i_, Q_3i_} versus matrix {O_3i_, P_3j_}, and relational degree Z_i_ versus Z_ij_. We used the adjacency list for the calculation.
b) *Second step: Filtering no-explicit-relationship author pairs*.
  The no-relationship author pairs (X_i_ = 0, Y_i_ = 0, Z_i_ = 0, and no cooperation) were filtered as {O_4i_, P_4j_} in the adjacency matrix and {L_4i_, Q_4i_} in the adjacency list, which formed the basic object in the subsequent analysis.
c) *Third step: Mining the relationship of A–C from {L_1i_, Q_1i_} {L_3i_, Q_3i_} {L_4i_, Q_4i_}*.
  {L_4i_, Q_4i_} was remarked as {A_k_, C_k_} (k stands for the number of author pairs) to find the D_k_ with the relations {A_k_–D_k_, C_k_–D_k_}. Looking for the synchronous relations with strength between A_k_ and D_k_, and C_k_ and D_k_ from {L_1i_, Q_1i_} {L_3i_, Q_3i_}, and matching the author pairs in {A_k_, C_k_}, the pseudocode is as follows:
  If one author in the pair of {A_k_, C_k_} = one author in a pair of {L_1i_, Q_1i_}, and another one in the pair of {A_k_, C_k_} = one author in a pair of {L_3i_, Q_3i_}, and another one in the pair of {L_1i_, Q_1i_} = another one in the pair of {L_3i_, Q_3i_}
  then the “one author in the pair of {A_k_, C_k_}” (so as the “one author in a pair of {L_1i_, Q_1i_}”) as C_α_, the “one author in a pair of {L_3i_, Q_3i_}” (so as the “another one in the pair of {A_k_, C_k_}”) as A_α_, and the “another one in the pair of {L_1i_, Q_1i_}” (so as the “another one in the pair of {L_3i_, Q_3i_}”) as D_α_ are marked.
  Finally, A_α_ and C_α_ could be connected according to D_α,_ and the final relationship strength of A_α_ and C_α_ would be the top value in all of the correlation scores (respectively equaling to the products of Y_k_ and Z_k_).
d) *Fourth step: Mining the relationship of B–D from {L_2i_, Q_2i_} {L_3i_, Q_3i_} {L_4i_, Q_4i_}*.
  {L_4i_, Q_4i_} was remarked as {B_k_, D_k_} (k stands for the number of author pairs) to find the A_k_ with the relations {A_k_–D_k_, A_k_–B_k_}. The synchronous relationship with strength between A_k_ and D_k_, and A_k_ and B_k_ were searched from {L_2i_, Q_2i_} {L_3i_, Q_3i_}, and the author pairs in {B_k_, D_k_} were matched. This process is similar to the process of A–C. Thus, the pseudocode was omitted. Finally, connected author pairs {B_β_, D_β_} with relationship strength X_k_ multiplied by Z_k_ could be acquired according to their co-connection with A_k_.
e) *Fifth step: Mining the relationship of B–C from {L_1i_, Q_1i_} {L_2i_, Q_2i_} {L_3i_, Q_3i_} {L_4i_, Q_4i_}*.
  The remaining (no relationship such as A–C and B–D) of {L_4i_, Q_4i_} were remarked as {B_k_, C_k_} (k stands for the number of author pairs) to find the A_k_ and D_k_ with the relationship {A_k_–D_k_, A_k_–B_k_, and C_k_–D_k_}. The synchronous relationship with strength between A_k_ and D_k_, A_k_ and B_k_, and C_k_ and D_k_ were searched from {L_1i_, Q_1i_} {L_2i_, Q_2i_} {L_3i_, Q_3i_}, and the author pairs in {B_k_, C_k_} were matched. The pseudocodes are as follows:
  If one author in the pair of {B_k_, C_k_} = one author in a pair of {L_2i_, Q_2i_}, and another one in the pair of {B_k_, C_k_} = one author in a pair of {L_1i_, Q_1i_}, and another one in the pair of {L_2i_, Q_2i_} = one author in the pair of {L_3i_, Q_3i_}, and another one in the pair of {L_1i_, Q_1i_} = another one in the pair of {L_3i_, Q_3i_}
  then marking the “one author in the pair of {B_k_, C_k_}” (so as the “one author in a pair of {L_2i_, Q_2i_}”) as B_χ_, “another one in the pair of {B_k_, C_k_}” (so as “the one author in a pair of {L_1i_, Q_1i_}”) as C_χ_, one author in the pair of {L_3i_, Q_3i_} (so as the “another one in the pair of {L_2i_, Q_2i_}”) as A_χ_, and another one in the pair of {L_1i_, Q_1i_} (so as the “another one in the pair of {L_3i_, Q_3i_}) as D_χ_
  Finally, B_χ_ and C_χ_ could be connected according to A_χ_ and D_χ_, and the final relationship strength of B_χ_ and C_χ_ would be the top value in all of the correlation scores (respectively equaling to the products of X_k_, Y_k_, and Z_k_).
  Thus far, all relationships among author pairs in {L_4i_, Q_4i_} had been established.

According to the above algorithm, potential relationships among no-direct-relationship core author set could be generated by the VBA program and Access databases. Finally, the comparison of new relationships and direct correlations (including CA, AC, ABC, ADC, and AKC) in 2011–2015 would be performed to identify the effectiveness of citation mining applied in the detection or promotion of more potential communications.

## Results and discussion

### Analysis results of AC, ABC, and ADC

**Fig 3:**
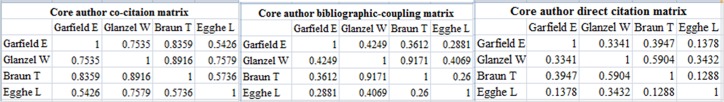
Normalized matrixes of tripartite citation analysis.

According to the tripartite citation analysis of AC, ABC, and ADC, we obtained three original relation matrixes and the corresponding normalized matrixes (Fig 3).

**Fig 4:**
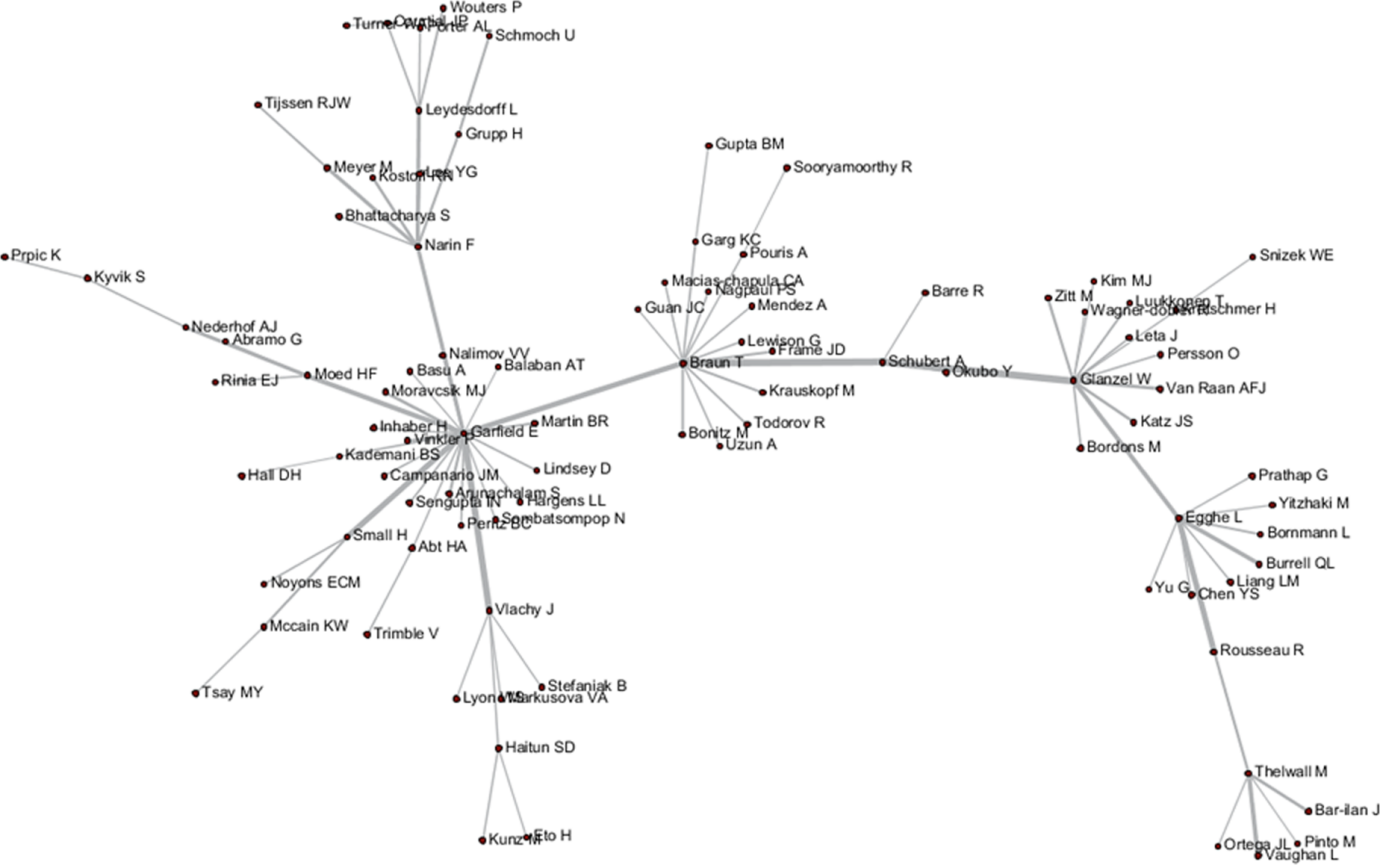
Author relationship network of AC.

**Fig 5:**
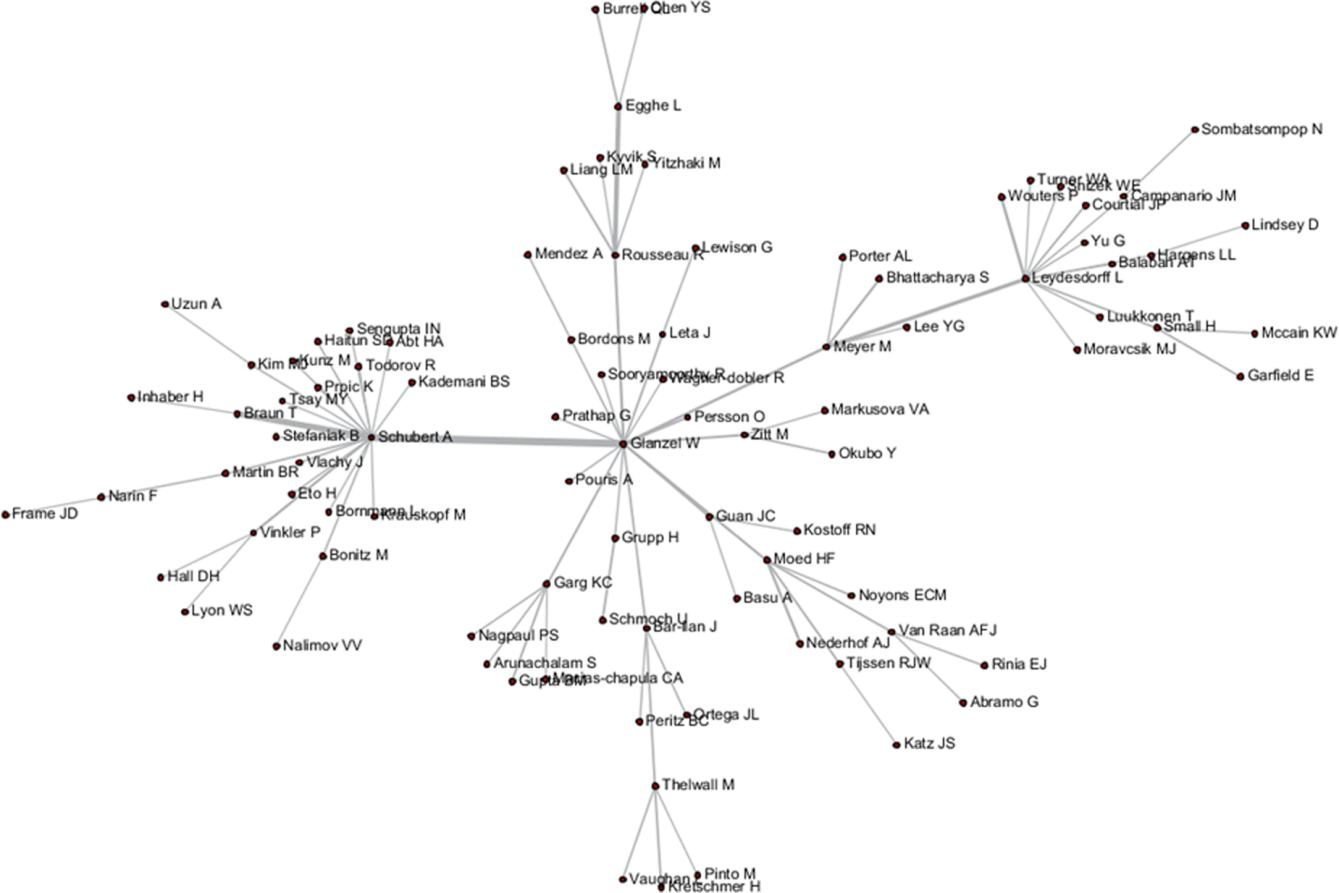
Author relationship network of ABC.

**Fig 6:**
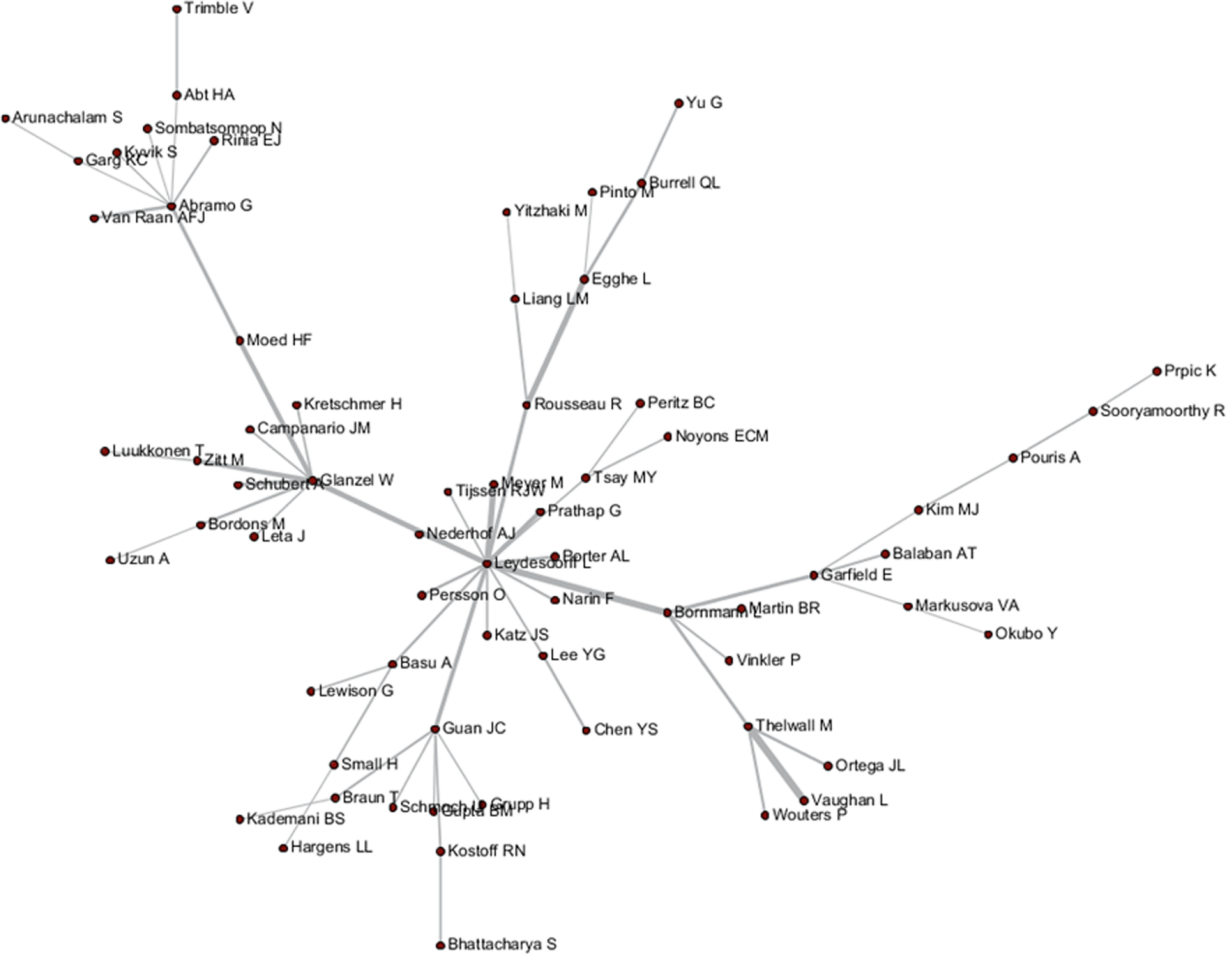
Author relationship network of ADC.

**Fig 7:**
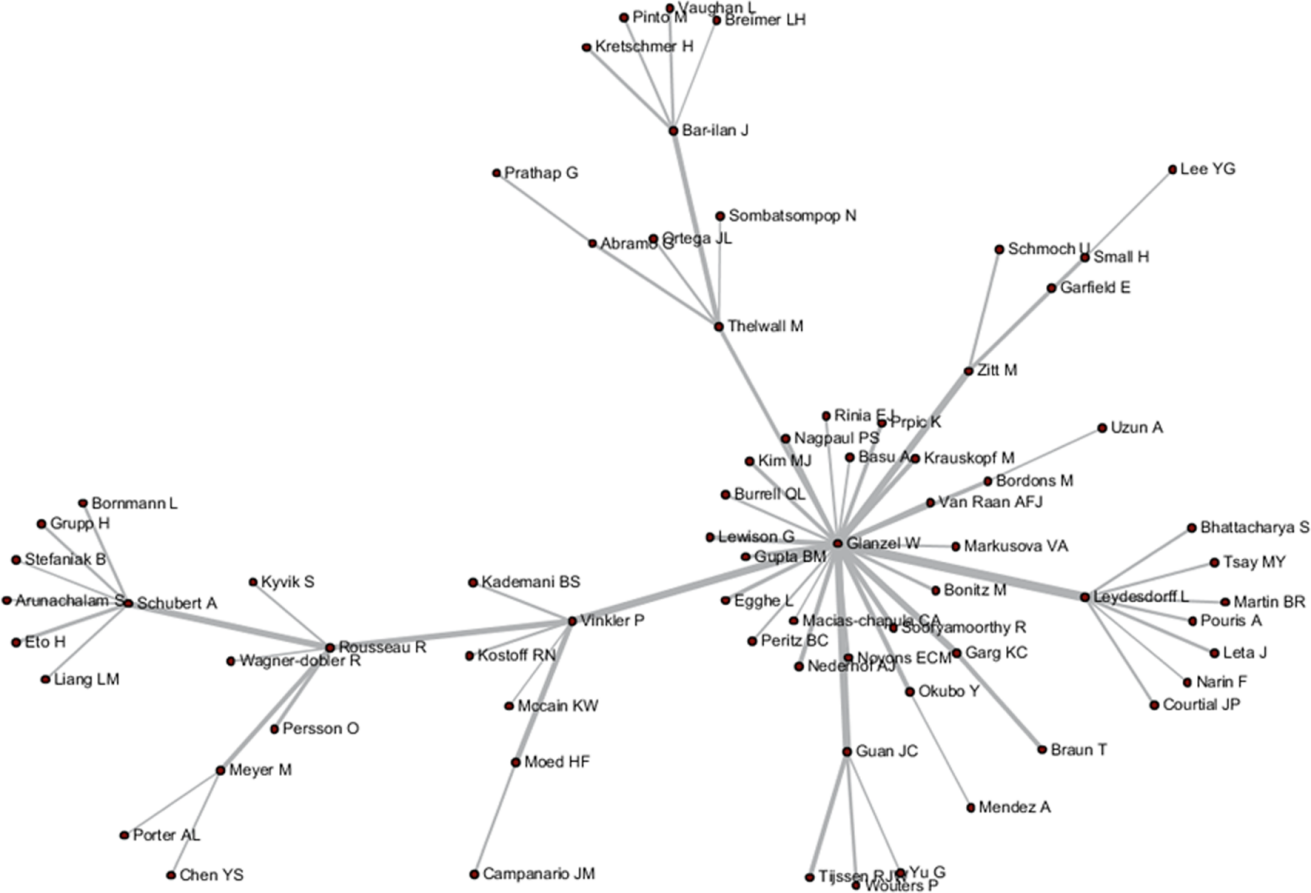
Author relationship network of AKC.

These tripartite matrixes and the AKC matrix could be visualized by the Network Workbench Tool (Figs 4–7).

The core nodes in each network are different, as shown in Figs 4–7. In the AC network, “(Moed HF, Narin F, Vlachy J)–Garfield E–Braun T–Schubert A–Glanzel W–Egghe L–Rousseau R–Thelwall M” are core associated scholars, all of whom form the main path in the network. In the ABC network, the main associated scholars include “Schubert A–Glanzel W–Meyer M–Leydesdorff L,” in which new core nodes, such as Garg KC, Bar-ilan J, Guan JC, and Zitt M, also emerge. In the ADC network, Leydesdorff L becomes the superior core node, and the associated path of “Abramo G–Glanzel W–Leydesdorff L–Bornmann L–Garfield E” becomes the main path. In the AKC network, “Schubert A–Rousseau R–Vinkler P–Glanzel W–Leydesdorff L” becomes the major associate scholars, and the key associations of Thelwall M, Guan JC, Zitt M, and Glanzel W are also reflected. These scholars are also at the heart of correlation formation among other scholars. Generally, the main connected path of “Garfield E–Schubert A–Glanzel W–Leydesdorff L” is more consistent in these four networks. However, the difference is also distinct, such that Garfield E is the main supporter for most core paths in the AC network, while the main supporter in the ABC and ADC networks are Glanzel W and Leydesdorff L, respectively. In view of the ADC revealing a more direct relationship, Leydesdorff L is more likely to be the builder of the potential connection.

As shown in Figs 4–7, author relevance was preliminarily identified by different citation methods. For example, *Leydesdorff L* has the strongest correlation with other authors in the direct citation networks, whereas the core correlation of the three types of indirect network (PCR) is relatively low, and some are even weak in the cooperative correlation. Meanwhile, *Narin F* presents the highest correlation degree in the AC network, and the correlation degree is comparatively lower in other networks. *Bornmann L* and *Sooryamoorthy R* are strongly correlated with the ADC and AKC networks, respectively. However, their correlations are low in other networks. In terms of partnership, these two authors do not establish any cooperative relationship with other co-authors. *Glanzel W* has the core link status and high centrality degree in all networks, followed by *Schubert A* and *Braun T*. In addition, *Bonitz M*, *Nagpaul PS*, *Mccain KW*, *Eto H*, *Stefaniak B*, *Krauskopf M*, *Wagner-Dobler R*, and *Macias-Chapula CA* have established indirect relationship in AC/ABC/AKC network. However, no direct relationship is observed in CA/ADC. Ucinet software was used to calculate the centrality measurement of the five types of network. The top ten authors are shown in Table 1. In Table 1, ND is the abbreviation of NrmDegree, which represents the normalization of degree.

**Table 1:** Author NrmDegree in the five networks (Top 10).

No.	Author	CA -ND	Author	ADC -ND	Author	AC -ND	Author	ABC -ND	Author	AKC -ND
1	Glanzel W	2.276	Leydesdorff L	8.215	Garfield E	11.583	Glanzel W	6.115	Glanzel W	14.982
2	Schubert A	2.126	Glanzel W	6.409	Glanzel W	9.588	Schubert A	5.928	Schubert A	14.504
3	Braun T	1.875	Bornmann L	4.602	Braun T	8.84	Leydesdorff L	3.599	Vinkler P	13.429
4	Rousseau R	0.475	Rousseau R	3.871	Schubert A	8.255	Rousseau R	3.361	Guan JC	13.429
5	Moed HF	0.375	Moed HF	3.613	Narin F	6.836	Braun T	3.326	Zitt M	13.166
6	Meyer M	0.275	Abramo G	3.226	Moed HF	6.705	Moed HF	2.714	Leydesdorff L	13.07
7	Van Raan AFJ	0.275	Egghe L	3.14	Leydesdorff L	6.276	Vinkler P	2.479	Rousseau R	12.545
8	Egghe L	0.25	Thelwall M	3.14	Egghe L	5.478	Zitt M	2.413	Garg KC	11.541
9	Kretschmer H	0.2	Guan JC	2.968	Van Raan AFJ	4.695	Meyer M	2.342	Moed HF	10.466
10	Liang LM	0.2	Zitt M	2.624	Small H	4.263	Guan JC	2.108	Sooryamoorthy R	14.982

### Results of PCR discovery

In this comparative study, AKC analysis was applied to produce the AKC matrix, in which the implementation process is similar with that of the ABC analysis (i.e., the authors are correlated with one another by indexing the same keywords). Table 2 presents the result of the QAP correlation test of CA, AKC, AC, ABC, and ADC matrixes. The results show that the correlation between the CA and ABC matrixes is the strongest, followed by the AC and ADC matrixes, and the AC and ABC matrixes. These findings indicate that the current study and the topic structure revealed by these three pairs are perhaps the most similar, or can be mutually complementary. In addition, the ABC matrix generally has the highest degree of correlations compared with all other relationship matrixes, which to some extent shows that the application of the ABC analysis can more accurately reveal the scientific structure of the disciplines. One of the possible reasons for using this analysis method is to divide into research groups and discover the subject structure by numerous scholars. This result supports the findings of Yan and Qiu [17–18], both of which revealed that ABC nearly has the highest similarity with other networks at the author level. Furthermore, the correlation coefficient of AKC and ABC matrixes is at a middle level, which indicates that AKC and ABC analyses share similarities to a certain extent. This finding is consistent with the conclusion of Yang [37].

**Table 2:** Correlations among the four matrixes according to QAP (* p<0.001).

QAP Correlations	AC	AKC	CA	ADC	ABC	AC′	AKC′	ABC′
AC	1	0.246*	0.47*	0.769*	0.644*	-	-	-
AKC	0.246*	1	0.139*	0.246*	0.437*	-	-	-
CA	0.47*	0.139*	1	0.479*	0.786*	-	-	-
ADC	0.769*	0.246*	0.479*	1	0.643*	-	-	-
ABC	0.644*	0.437*	0.786*	0.643*	1	-	-	-
AC′	-	-	-	-	-	1	0.143*	0.374*
AKC′	-	-	-	-	-	0.143*	1	0.54*
ABC′	-	-	-	-	-	0.374*	0.54*	1

Excluding ACR connections (including CA and ADC) from PCR (including AC, ABC, and AKC), the matrixes of AC′, ABC′, and AKC′ were obtained. The QAP correlation test for the new three matrixes was performed, with results shown in Table 2. The two groups of correlation strengths in successive QAP results were compared (marked in color red). The comparison shows that the relation degrees among AC′, ABC′, and AKC′ could also maintain significant correlations, especially AKC′ and ABC′, which share higher relevancy than the original matrixes. This condition indicates that these authors connected by PCR are likely to produce academic exchanges based on similar themes.

The three new matrixes with ACR connections from 2011 to 2015 were further compared in Table 3. According to the QAP analysis, the correlation coefficient between the new ACR (after 2011) and the old PCR (before 2011) is 0.225 (p<0.001). This result can also sustain the assumption about applying PCR in the detection of new academic exchanges. We converted the new relationships into author pairs and analyzed them with Pearson correlation. We found that the three previous relationships showed a more apparent correlation with the new PCR and ACR. Among those relationships, ABC has the strongest correlation and the highest predictability. In addition, the previous and new PCRs are consistent, and the correlation between the new PCR and ACR is significant. Meanwhile, ABC also reflects the highest correlation, followed by AC.

**Table 3:** Correlations among matrixes between two sets (before and after 2011) according to the Pearson coefficient.

Pearson correlation		Before 2011			After 2011				
		AC′	ABC′	AKC′	AC″	ABC″	AKC″	ACR″	PCR″
Before 2011	AC′	1	0.374*	0.143*	0.492*	0.091*	0.036	0.167*	0.453*
	ABC′	0.374*	1	0.540*	0.360*	0.301*	0.052*	0.229*	0.398*
	AKC′	0.143*	0.540*	1	0.239*	0.298*	0.180*	0.205*	0.292*
After 2011	AC″	0.492*	0.360*	0.239*	1	0.338*	0.136*	0.446*	0.964*
	ABC″	0.091*	0.301*	0.298*	0.338*	1	0.290*	0.529*	0.576*
	AKC″	0.036	0.052*	0.180*	0.136*	0.290*	1	0.143*	0.200*
	ACR″	0.167*	0.229*	0.205*	0.446*	0.529*	0.143*	1	0.537*
	PCR″	0.453*	0.398*	0.292*	0.964*	0.576*	0.200*	0.537*	1

* p<0.001

Further analysis of the author pairs before and after 2011 demonstrates that several author pairs have strong PCR correlations, such as *Bordons M*–*Glanzel W*, *Katz JS*–*Leydesdorff L*, and *Braun T*–*Rousseau R*. In addition, the new ACR correlations appeared after 2011, which suggests that the PCR relationship promotes the occurrence of the ACR relationship. The new main ACR author pairs are listed in Table 4.

**Table 4:** Discovery of author pairs with both relatively higher ACR″ and PCR′.

Author pairs	Before 2011			After 2011	
	AC′	ABC′	AKC′	ACR″ (= ADC″)	PCR″
Bordons M–Glanzel W	84	40	5	7	101
Thelwall M–Wouters P	3	7	1	6	35
Basu A–Small H	2	2	1	4	14
Bornmann L–Guan JC	5	2	5	4	26
Katz JS–Leydesdorff L	45	7	0	4	46
Leydesdorff L–Liang LM	1	16	3	4	47
Braun T–Rousseau R	55	30	3	3	42
Glanzel W–Guan JC	17	75	14	3	40
Guan JC–Zitt M	6	32	8	3	22
Leydesdorff L–Tsay MY	15	10	6	3	27
Noyons ECM–Tsay MY	4	2	1	3	0
Porter AL–Zitt M	4	12	1	3	37
Egghe L–Kretschmer H	16	7	3	2	76
Moed HF–Persson O	33	18	2	2	22

### Results of author relationship mining

**Fig 8:**
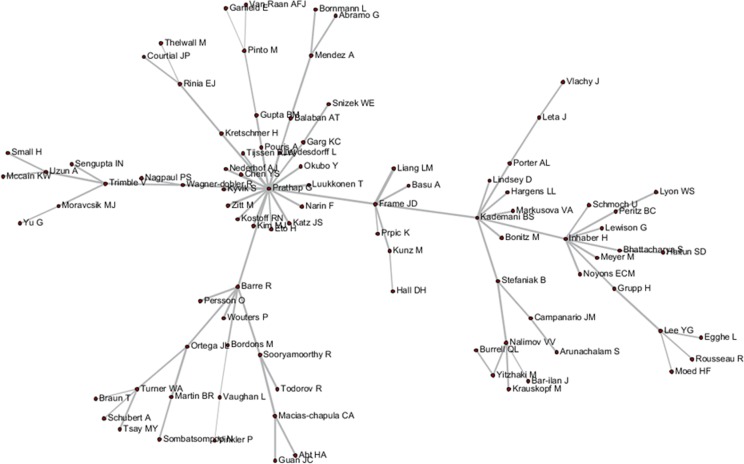
Author relationship network of A–C.

**Fig 9:**
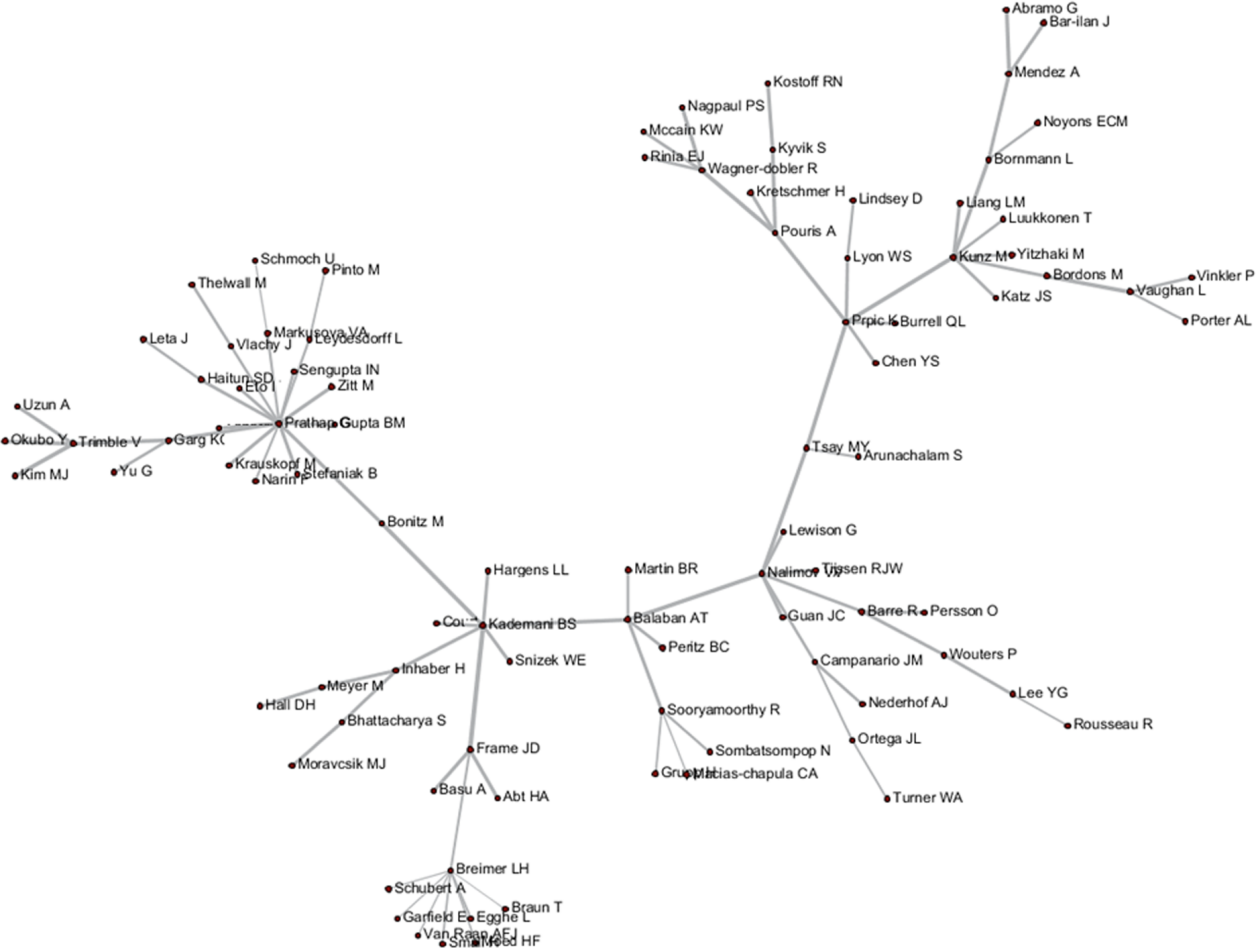
Author relationship network of B–D.

**Fig 10:**
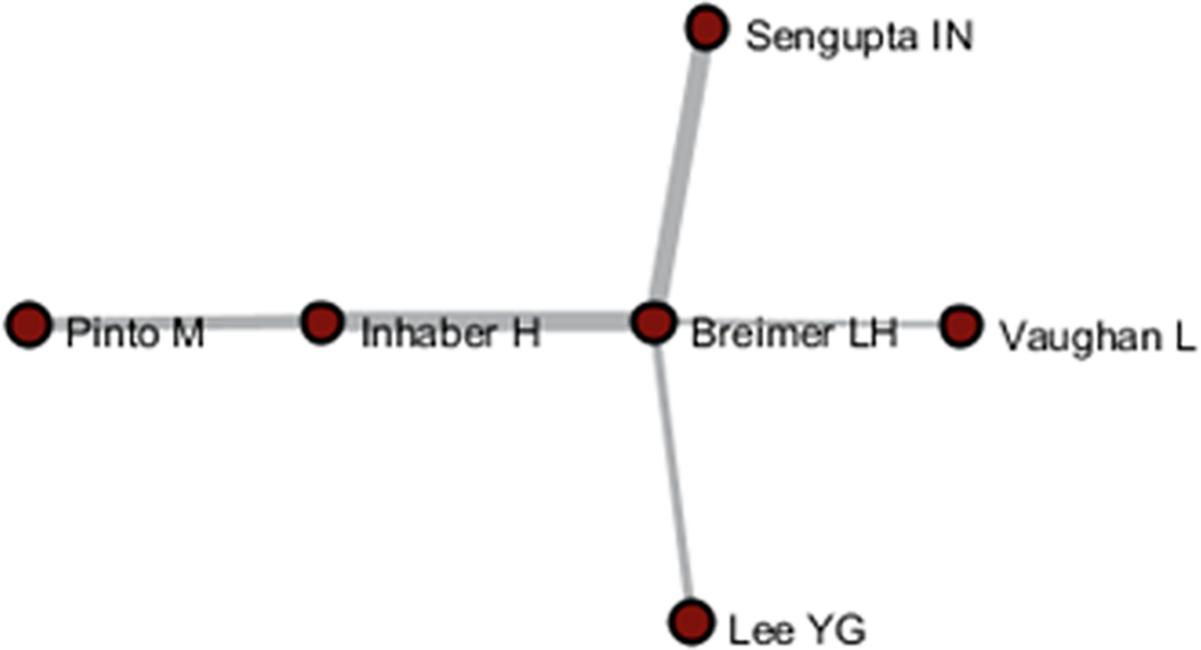
Author relationship network of B–C.

According to various analyses of AC, ABC, ADC, and CA, we found that *Glanzel W* had direct relationship with others, whereas most of the core authors have not related to all of the other authors. Following the five steps described above, new relationships between Authors A and C, B and D, and B and C were discovered, with respective author relation pairs: 1,793, 1,916, and 10. Subsequently, the final results among A–C, B–D, and B–C were acquired and visualized by the Network Workbench Tool, as shown in Figs 8–10. It is needed to point out that although the networks generated by PathFinder are sparse (in this network, only core node and connections are preserved, in order to concentrate on crucial points), the identification of the implicit associated author pairs is based on all the network data. Therefore, the relational mining process is still valid in this finite data set.

As shown in Figs 4–6, *Pinto M*, *Lee YG*, *Prathap G*, and *Breimer LH* et al. had few direct relationships with others, and even rarer relationship among them. In particular, *Breimer LH*, whose research focused on interdisciplinary fields, such as medical and information sciences, had no co-citation relation with the core authors in the field of scientometrics, and few direct-citation and bibliographic-coupling relations with others. However, in the relation mining of “A–C” by joining ABC with ADC, indirect relationships between *Breimer LH* and 77 authors were established, and *Breimer LH* became a core node in the A–C network to replace the independent node in the AC network and peripheral node in other networks. Meanwhile, *Prathap G*, who was occupied with interdisciplinary research between material and information sciences, established indirect relationships with approximately 60 authors. *Pinto M*, an emerging scholar in scientometrics (with 5 papers and 10 citations in the dataset), has not yet formed a certain influence in the field. Few direct relations are observed between *Breimer LH* and elder statesmen in the field. However, he was also supposed to be the core node in A–C and B–D networks.

The first direct relationship mining among *Breimer LH*, *Inhaber H*, *Lee YG*, *Sengupta IN*, *Vaughan L*, and *Pinto M* was not fully achieved. Thus, the mining of “B–C” joining ABC, ADC, and AC were conducted with the newly discovered direct relationships. As shown in the results presented in Fig 10, the core link status of *Breimer LH* was re-verified, and his research can be considered gaining more attention from colleagues and that more communication and linkages are established over time because of him.

To verify the existence of author relationship mining based on tripartite citation analysis proposed in this study, correlation analysis between the mining results and author direct relationship status (such as co-author and co-citation) was recently performed to reveal the predictive and practical value of the mining method and results. New AC, ABC, ADC, AKC, and CA matrixes from 2011 to 2015 have been investigated; and five matrixes, including CA, AC, ABC, ADC (symmetrized), and AKC were developed. Four author pairs could be identified according to the comparison of data mining before 2011(A–C and B–D), as well as evident relationship after 2011 (AKC″, CA″, AC″, ADC″, and ABC″), which are shown in Table 5. Although no co-authorship exists among these author pairs, the other direct relationships, such as AKC, ADC, and ABC, are still evident, especially *Leydesdorff L* and *Prathap G*.

**Table 5:** Author pairs mined by A–C and B–D compared with apparent relationships after 2011.

Author Pairs	Before 2011		After 2011				
	A–C	B–D	AKC″	CA″	AC″	ADC″	ABC″
Leydesdorff L–Prathap G	0.72	0.64	9	0	69	14	30
Prathap G–Zitt M	0.75	0.73	2	0	3	0	9
Narin F–Prathap G	0.72	0.62	1	0	3	0	0
Kostoff RN–Prathap G	0.65	0.69	1	0	4	0	0

**Fig 11:**
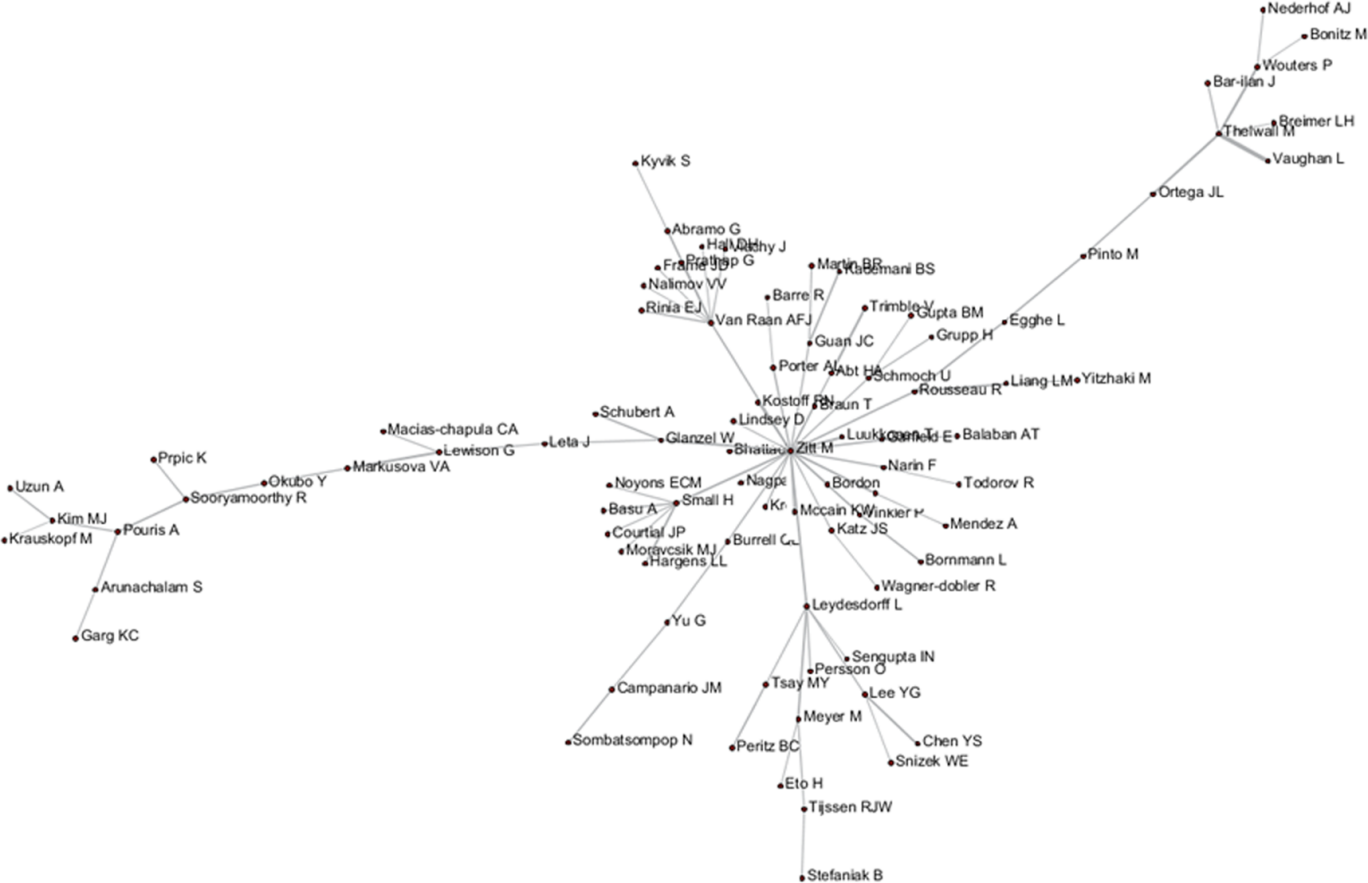
Author CC network in 2011 to 2015.

On the normalization process, given the presence of large amounts of 0 module caused by less amount of data within a limited time, another type of standardized method was selected. The AC matrix was considered as an example; the co-citation frequency between Authors A and B is x, the total frequency of A co-cited with all authors is m, the total frequency of B co-cited with all authors is n, and the correlation strength between Authors A and B is x/m+x/n. This analogy indicates that standardized matrixes of CA, AC, ABC, and ADC were obtained. Finally, a comprehensive correlation (CC) matrix was developed by adding four types of correlation values. Pearson correlation test was performed among author pairs of A–C, B–D, CC, and AKC, which correspond to two types of data sets, namely, A–C and B–D. The results are shown in Table 6, and the CC matrix was visualized using the Network Workbench Tool (Fig 11). Network Scaling MST-PathFinder was performed to show the network clearly. Therefore, the previously revealed correlations are not fully displayed.

**Table 6:** Pearson correlation results among the four types of author pairs.

Pearson Correlation	A–C	AKC	CC	Pearson Correlation	B–D	AKC	CC
**A–C**	1	0.075	0.013	**B–D**	1	0.091	−0.124
**AKC**	0.075	1	0.31*	**AKC**	0.091	1	0.305*
**CC**	0.013	0.31*	1	**CC**	−0.124	0.305*	1

* p<0.001

As shown in Table 6, certain degrees of positive correlation are observed among A–C, AKC, and CC, and B−D and AKC, which could indicate that the indirect relationships mentioned above would turn into direct relationships to some extent in the near future. Scholars in the field have consciously or unconsciously paid close attention to or cite links (including co-cited, coupled, and citing) with other scholars who shared indirect relationships instead of the direct relationships with the former authors, and even produced substantial cooperation among one other. Notably, the correlation between the AKC and CC matrixes is relatively significant. This result can be compared with previous results shown in Tables 2 and 3, in which AKC is also significantly correlated with others, even though the related values are comparatively lower (except for ABC). Therefore, AKC analysis may help in revealing the evolution of the existing relationships. Meanwhile, the relationship mining method proposed in this study could aid in revealing unknown relationships that complement with AKC analysis or other methods, such as topic analysis.

## Conclusions

Various relationships exist in academic networks, such as CA, AC, ABC, ADC, and AKC. In a given field, the intensity and the associated attributes among scholars may exhibit significant differences in terms of the different network correlations. Some scholars showed strong ACR correlations, while some had key positions in PCR association. In this study, we compared the five types of matrixes by QAP and found that ABC has the nearly highest similarity with other networks. This finding can demonstrate the superiority of ABC analysis in revealing an academic community and its scientific structure. Furthermore, the correlation coefficient of ABC and AKC is higher than the coefficients among AKC and others, indicating that AKC and ABC can be complementarily applied in potential communication mining.

By comparing the relationship of ACR and PCR, a particular phenomenon was observed, in which only PCR existed among scholars without ACR in the field of scientometrics, such as among *Bonitz M*, *Nagpaul PS*, *Mccain KW*, and *Eto H*. By analyzing PCR with the ACR correlations excluded (i.e., including AC′, ABC′, and AKC′) by QAP, we found that the relationship degrees among AC′, ABC′, and AKC′ can also maintain significant correlations, especially AKC′ and ABC′, which share higher relevancy than the original matrixes. This result indicates that these authors connected by PCR are likely to produce more academic exchanges and scientific innovations because of similar themes rather than social attribute association (e.g., teacher–student or co-worker relationships).

By conducting Pearson correlation analysis, the case study confirmed that a significant correlation existed between the PCR that appeared before 2011 and the new ACR″ and PCR″, which appeared after 2011. Furthermore, continuity existed between PCR′ and PCR″, and the associated relationship between ACR″ and PCR″ were also significant. Particularly, ABC′, ABC″, and other relations have been highly correlated, which indicated that ABC analysis shows a good application potentiality in relationship prediction and discovery to some extent and may reflect actual communication.

On the basis of the algorithm design and the empirical analysis, the deduction from the analysis results from AC, ABC, and ADC to the potential author relationships mining is probable and practicable. For example, the relationship between *Leydesdorff L* and *Prathap G* revealed by A–C/B–D in the case study achieved a high degree of correlation in the practice after 2011 (including AC, ADC, and ABC, which did not exist before 2011). The author correlation between *Breimer LH* and *Vaughan L* obtained by two-time mining was also consistent with the new correlation in 2011, which once again confirmed the validity and the potential value of the proposed method for revealing author relationships.

The results presented above revealed that the indirect relationships among interdisciplinary scholars or novice researchers can be mined by the method combined with tripartite citation analysis, which helps with specific scientific cooperation and broader communication. In comparison with the direct relationship presented recently by Pearson correlation, the author mining method proposed in this study helps in revealing unknown relationships and could complement with AKC analysis or other methods, such as topic analysis. These methods could be applied in discovering research fellows, exploring potential partners, as well as tracking scholars with related research and their research direction.

In conclusion, this study attempted to discover PCRs. Through the correlations between the measurements, the proposed method could be used to explain that the establishment of co-citation or coupling relation may promote the production of the actual communications. This finding suggests that these two relations could identify potential collaboration partners for both individuals and teams. The proposed author relationship mining method based on tripartite citation analysis could also be an effective method for discovering future relationships among scholars and promoting scientific communication and innovation development.

In addition, we recognized the existence of limitations in the dataset of “core authors” by selecting only the first author as citation data and defining the threshold of the publication number and citation frequency. We performed such step despite the fact that only the first cited authors tallied in the database of WOS and regardless of the difficulty of a specific time window for obtaining a sufficient linking signal (e.g., the data in the first period of 1978–2011, which was acquired in 2012, is difficult to be regained at present). However, this paper was an attempt to propose an idea and process in author relationship mining in the context of five types of scholarly networks; thus, the collection of core authors targeted by this research was supposed to be useful in the application of relationship mining method. Nevertheless, we are faced with the data limitation, thus the need to present a more credible empirical study with a sizable sample and enhance the practicality and effectiveness in PCR discovery by tripartite citation analysis. Finally, as an attempt, the proposed method should also be applied in various fields. However, the method was tested only in the field of scientometrics due to computational complexity, the amount of data obtained, and so on. In addition, some of the studies exhibit positive results, which are applicable only in the field of scientometrics [47]. In the context of scientometrics, the results are easier to explain and rigorously confirmed. In further research work, the proposed method should be applied in other fields to further confirm its effectivity and rationality.

## Supporting information

S1 Table AC original matrix in period of “Before 2011”. (XLSX)

S2 Table ADC original matrix in period of “Before 2011”. (XLSX)

S3 Table ABC original matrix in period of “Before 2011”. (XLSX)

S4 Table CA original matrix in period of “Before 2011”. (XLSX)

S5 Table AKC original matrix in period of “Before 2011”. (XLSX)

S6 Table AC original matrix in period of “After 2011”. (XLSX)

S7 Table ADC original matrix in period of “After 2011”. (XLSX)

S8 Table ABC original matrix in period of “After 2011”. (XLSX)

S9 Table CA original matrix in period of “After 2011”. (XLSX)

S10 Table AKC original matrix in period of “After 2011”. (XLSX)
